# Exploring the Impact of IGC‐AD1 on Serum Potassium Levels in Alzheimer’s Disease: Insights from a Phase I Trial

**DOI:** 10.1002/alz.095358

**Published:** 2025-01-09

**Authors:** Laura Delgado‐Murillo, Maria Alejandra Tangarife, Evelyn Gutiérrez, Fabio Camargo, María Paula Naranjo, María Margarita Venegas, Laura Tatiana Sánchez, María Juanita Arbeláez, Saadia Shahnawaz, Varduhi Ghazaryan, William Julio De Jesus, Elliot Hong, Claudia Grimaldi, Jagadeesh S Rao, Ram Mukunda

**Affiliations:** ^1^ IGC Pharma, Bogotá, Bogotá D.C. Colombia; ^2^ IGC Pharma, Potomac, MD USA; ^3^ Santa Cruz Behavioral, Bayamon, PR USA; ^4^ UTHealth Houston McGovern Medical School, Houston, TX USA

## Abstract

**Background:**

This study investigates the impact of IGC‐AD1, a combination comprising of low concentration of delta‐9 tetrahydrocannabinol (“THC”) and melatonin on blood serum potassium levels in patients with Alzheimer’s disease (“AD”). Loss of intracellular compartmentalization of potassium is a characteristic of AD pathology, with supporting studies indicating significantly lower potassium levels in intracellular compartments of AD brains and an associated increase in serum potassium levels in AD subjects. Here, we present preliminary safety lab data from a Phase I trial of AD patients administered with IGC‐AD1.

**Methods:**

Thirteen Puerto Rican AD patients (mean age: 80.18 ± 6.22 years, 70% women) participated in a three‐cohort Phase I trial. Cohorts 1, 2 and 3 received 1 mL of IGC‐AD1 once a day, twice a day, or thrice a day for 14 days (“EOT”) with a washout period between each cohort. Only Cohort 1 data were analyzed. Blood draws occurred at baseline, day 5, day 10, and day 14.

**Results:**

The analysis showed a statistically significant decrease in potassium serum levels from baseline (4.38 mmol/L) to EOT (4.16 mmol/L) in Cohort 1 on active medication (p = 0.013), while no differences were observed in patients on placebo (baseline: 4.65 mmol/L and EOT: 4.7 mmol/L; p = 0.874).

**Conclusions:**

Future imaging studies could explore the use of radioactive potassium tracers for diagnosis. The study provides insights into a) potassium as a potential blood‐based diagnostic biomarker for AD and b) the potential of IGC‐AD1 as a disease‐modifying medication, given the preliminary data showing a reduction in serum potassium levels in AD subjects post‐treatment.

